# Combined HIIT and Resistance Training in Very Long-Chain Acyl-CoA Dehydrogenase Deficiency: A Case Report

**DOI:** 10.3389/fphys.2019.00650

**Published:** 2019-05-28

**Authors:** Alba M. Herrera-Olivares, Jose A. Fernández-Luque, Carmen Paradas, Alejandro Lucia, Alfredo Santalla

**Affiliations:** ^1^Faculty of Sport Sciences, Universidad Europea de Madrid, Madrid, Spain; ^2^Faculty of Sport Sciences, Universidad Pablo de Olavide, Seville, Spain; ^3^Neuromuscular Disorders Unit, Department of Neurology, Instituto de Biomedicina de Sevilla, Hospital Universitario Virgen del Rocío, CSIC-Universidad de Sevilla, Seville, Spain; ^4^Biomedical Network Research Centre on Neurodegenerative Diseases (CIBERNED), Madrid, Spain; ^5^Instituto de Investigación Hospital 12 de Octubre (i+12), Madrid, Spain

**Keywords:** VLCADD, training, exercise, neuromuscular disorders, FATmax

## Abstract

Very long-chain acyl-CoA dehydrogenase deficiency (VLCADD) is a rare disorder of mitochondrial fatty acid β-oxidation characterized by a spectrum of clinical manifestations. Patients with the adult-onset form can present with muscle pain, rhabdomyolysis and myoglobinuria after physiological stress, such as fasting and exercise. We report on a 23-year-old female patient with a history of recurrent rhabdomyolysis. The patient completed a 6-month supervised combined (high-intensity interval training [HIIT] + resistance training) program, with the addition of a medium chain triglyceride + carbohydrate supplement provided 60 min before each session. The HIIT consisted of 6 sets of 70–80 s performed at maximum intensity with a minimum cadence of 100 rpm. Resistance training consisted of a circuit of basic exercises with dumbbells and elastic bands, with sets of 4–7 repetitions. The patient was evaluated at months 0, 3 and 6 using an incremental discontinuous step protocol, with steps of 1 min of exercise/1 min of passive recovery, at a high pedal cadence. The test started at 10 W, with a load increase of 10 W/step. Blood creatine kinase (CK) concentration was measured before each evaluation. There was a training-induced increment of 90.2% in peak oxygen uptake (VO_2peak_), 71.4% in peak power output and 24.7% in peak heart rate. The patient reported no muscle pain, contractures, rhabdomyolysis (basal CK concentration was always <200 U/L) or hospital admissions during the training period. After completion of 6-month program, the patient remained active, doing similar but non-supervised training for 1.5 years (to date). During this period, the patient has not reported myalgias, contractures, rhabdomyolysis or hospital admissions. Our preliminary data suggest that it is possible to carry out a combined (HIIT + strength) training program in patients with VLCADD, safely (without muscle contractures or rhabdomyolysis) and obtaining high values of VO_2peak_ and cycling power output.

## Introduction

Very long-chain acyl-CoA dehydrogenase (VLCAD) deficiency (VLCADD, OMIM 201475) is an autosomal recessive disorder (Andresen et al., 1999; Wanders et al., 1999; Lindner et al., 2010) with an estimated incidence of 1 in 30,000–100,000 newborns (Tucci et al., 2014). VLCAD is a mitochondrial membrane enzyme that catalyzes the dehydrogenation of very long chain fatty acids (14–20 carbons) (Andresen et al., 1999), which is the first step in the β-oxidation of fatty acids (Leslie et al., 1993). VLCADD is a clinically heterogeneous disease with three major phenotypes: (i) a severe infantile form with early onset and high mortality, which manifests with cardiac arrhythmia, cardiomyopathy and liver disease; (ii) a milder childhood form with late onset, which typically presents with hypoketotic hypoglycemia but with rare cardiomyopathy and low mortality; and (iii) an adult form with isolated involvement of the skeletal muscle that is commonly accompanied by rhabdomyolysis and myoglobinuria (Andresen et al., 1999; Arnold et al., 2009).

The most common form of VLCADD is the adult manifestation, and treatment consists mainly of dietary advice aimed at preventing catabolism, including following a diet rich in carbohydrates (CHO), low in very long chain fatty acids and supplemented with medium-chain triglycerides (MCT) (Arnold et al., 2009; Spiekerkoetter et al., 2009). It is also recommended that patients with VLCADD avoid prolonged exercise and fasting (Voermans et al., 2005; Diekman et al., 2016; Bleeker et al., 2018) to prevent rhabdomyolysis and myoglobinuria (Merinero et al., 2018). However, even following these recommendations, patients can still experience exercise intolerance, myalgia and, occasionally, episodes of rhabdomyolysis (Diekman et al., 2016), which is a serious complication (Hisahara et al., 2015) and can cause kidney damage and even kidney failure (Laforêt et al., 2009).

Perhaps for these reasons, there has been only one study examining the bioenergetic responses to aerobic exercise in patients with VLCADD (Diekman et al., 2016). Patients performed 45 min of exercise at a constant load corresponding to the maximal fat oxidation (FATMAX) (38 ± 4% of peak oxygen uptake [VO_2peak_]), of which the last 5 min were performed inside a magnetic resonance scanner. Results showed an impairment in energetic muscle balance, including a decrease in phosphocreatine and an increase in inorganic phosphate concentrations, without changes in plasma acyl-carnitine levels. Based on these results, the authors hypothesized that this energy crisis during exercise (insufficient ATP turnover) was responsible for the observed rhabdomyolysis (Diekman et al., 2016).

Accordingly, increasing ATP synthesis capacity during exercise through a training program could be an effective strategy to reduce the symptomatology of VLCADD (exercise intolerance, myalgia and risk of rhabdomyolysis). However, to our knowledge, no study has analyzed the effects of exercise training in VLCADD patients. Here we tested the applicability of combined (high-intensity interval training [HIIT] + strength training) exercise program in a young VLCADD patient.

## Case Report

**FIGURE 1:**
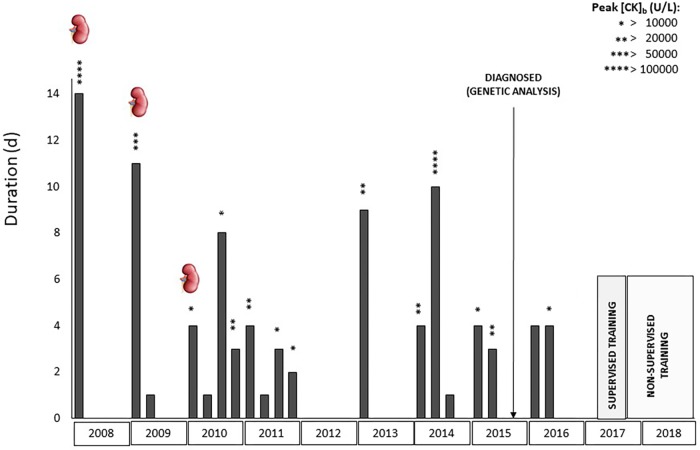
Clinical history of the patient from her first hospital admission to date. Each bar represents a hospitalization, detailing its duration (days) and highest concentration of creatine kinase (CK) reached. Kidney Symbol: renal failure.

The patient was a 23-year-old woman (height: 167 cm, weight: 72.3 kg) from a non-consanguineous family, with asymptomatic parents and brothers. From 14 years of age she had 19 episodes of rhabdomyolysis, all of them requiring hospitalization (two of them after the genetic diagnosis), with a median duration of 4 days (range 1–14), and a maximum creatine kinase (CK) concentration of 39,994 ± 66,148 U/L (range 2,121–276,000 U/L). Three episodes of rhabdomyolysis were accompanied by renal failure (Figure 1). Physical examination and CK levels were normal between the episodes. At 21 years of age, the patient was assessed using a targeted next-generation sequencing-based panel containing 256 neuromuscular disease genes, and found to have a compound heterozygous mutation c.589G > A (p.Val197Met)/c.1742T > C (p.lle581Thr) in the gene (*ACADVL*, MIM 609565) encoding VLCAD. The patient gave her written consent to participate in the study and for the data to be published, after a thorough explanation about VLCADD and the purpose of the study, which was approved by the local institutional ethics committee.

### Laboratory Evaluation

At the first visit to our laboratory (March 2017), the patient underwent ergospirometry. It was our intention to use a stepped incremental ergospirometry protocol with an initial power of 0 watts and a power increase of 30 watts/3 min, at 60 rpm of pedaling rate (Diekman et al., 2016), but the patient developed muscle pain and was unable to complete the 1st step (0 watts). Thus, she was asked to maintain a high pedaling rate (∼100 rpm) with the aim of recruiting type IIA and IIX fibers (not dependent on fatty acids) (Vollestad and Blom, 1985). As the patient reported no pain at these pedaling cadences, a test was performed on an electromagnetic cycle ergometer (Ergometrics 900, Ergoline, Barcelona, Spain) using an incremental discontinuous step protocol, with steps of 1 min of exercise interspersed with periods of 1 min of passive recovery, always maintaining a high cadence. The test was started at 10 watts, with a load increase of 10 watts/step. At the end of each step, a rating of perceived effort (RPE) was assessed according to the OMNI scale (Utter et al., 2004), and lower limb pain perception was scored using a numeric rating scale (NRS) (0–10) (Hawker et al., 2011). She was also asked at the end of the test whether she had a perception of muscle crises. The test was performed in the exercise physiology laboratory of Pablo de Olavide University under medical supervision, and the intensive care unit of Hospital Virgen del Rocío (Seville, Spain) was advised to be prepared.

Heart rate (HR, in bpm) was continuously monitored during the test from a 12-lead ECG. A breath-by-breath automatic system (CPX ultima, Medical Graphics Corporation, St Paul, MN, United States) was used to measure gas-exchange parameters: oxygen uptake (VO_2_, in mL⋅min^−1^ and mL⋅kg^−1^⋅min^−1^), carbon dioxide production (VCO_2_, mL⋅min^−1^), ventilation (VE, in L⋅min^−1^), and ventilatory equivalent for O_2_ (VE⋅VO_2_^−1^). Average values of the last 10 s of each step were obtained.

One hour before evaluation the patient ingested 500 mL of an isotonic drink (30 g CHO) Gatorade, PepsiCo, Purchase, NY, United States) with 15 g of MCT (Myprotein, Cheshire, United Kingdom), proportions that have been recommended for intestinal absorption (Jeukendrup et al., 1998; Jeukendrup, 2014). It is known that MCT can circumvent the block in the β-oxidation of long-chain fatty acids in VLCADD and can provide an alternative energy substrate to long-chain triglycerides (LCT) (Shiraev and Barclay, 2012), in addition to decreasing the oxidation of CHO, and reducing the risk of lactic acidosis induced by exercise (Behrend et al., 2012). Moreover, supplementation with CHO increases blood glucose levels and improves performance (Tsintzas et al., 2000). Accordingly, the role of this supplement was to (i) increase the work capacity during the test, (ii) maintain the glycemia level to reduce fat oxidation during recovery, and (iii) provide MCT for oxidation during the hours after assessment, thereby reducing the risk of post-exercise rhabdomyolysis (Diekman et al., 2016). Blood samples were collected 2 days before each evaluation to measure CK concentration. Laboratory evaluations were repeated at the end of the 3rd and 6th month.

### Training Program

**FIGURE 2:**
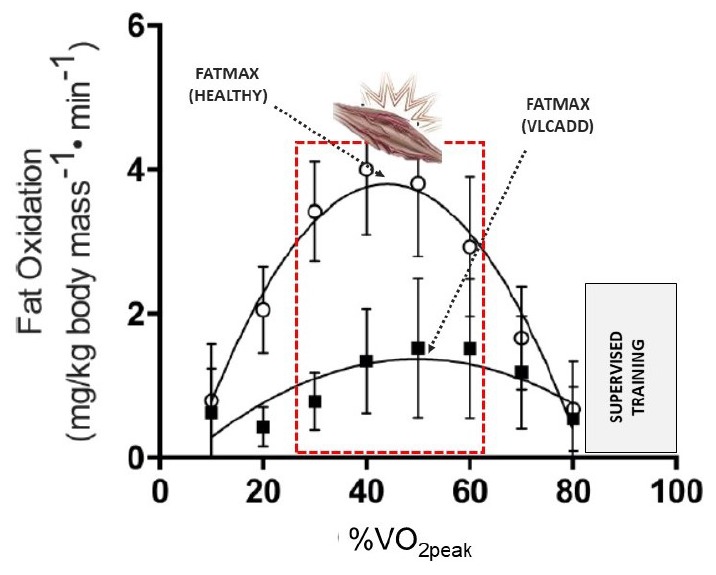
Fat oxidation capacity in exercise in a healthy person vs. a patient with VLCADD. Muscle discomfort during exercise appears at intensities close to FATMAX (marked in red), modified from (10). High-intensity interval training was always at high intensity (in which the demand for fat oxidation is practically null). Completed only 40 s of the last step.

The patient followed a 6-month combined (HIIT + strength training) exercise program supervised by a fitness specialist. The first month consisted of only HIIT (2 days⋅week^−1^), while in the following 5 months combined training was carried out 4 days⋅week^−1^ (HIIT 2 days⋅week^−1^ and strength training 2 days⋅week^−1^). HIIT was done in cycle ergometer and consisted of 6 sets of 70–80 s performed at maximum intensity with a minimum cadence of 100 rpm, with 1 min passive recovery periods between sets. We chose this type of training to maintain a high demand for glycolysis during exercise and, therefore, avoid the dependence of lipolysis on energy production (at the highest possible intensity, well above FATMAX) (Figure 2). HR and RPE were registered at the end of each set, while pain perception was registered at the end of the training session (Hawker et al., 2011). Three hours later, the patient was asked if she had a perception of (i) muscle swelling similar to that experienced before her hospital admissions (in order to anticipate a possible muscle crisis) and (ii) risk of actual muscle “crisis” (i.e., rhabdomyolysis).

The following strength training exercises were performed with dumbbells and elastic bands: bench press, biceps curl, bent-over dumbbell row, lying triceps extension and dumbbell lateral rise, and also ½ squat using body weight. The exercise routine was designed as a circuit, with 2 laps of 4–7 repetitions, with 2 min rest between sets and 4 min rest between laps. The OMNI Resistance Exercise Scale (Robertson et al., 2003) was used to measure RPE. The initial load was fixed at 5–7 of this 0–10 scale. Three hours after each strength session the patient was asked about muscle swelling. The morning after each session, she was asked if she had delayed-onset muscle soreness (using a scale of 0–10 in case of presentation).

Given the high intensity of HIIT, and the excess post-exercise oxygen consumption (EPOC) elicited by intense exercise bouts (McGarvey et al., 2005; Knab et al., 2011; Shiraev and Barclay, 2012; Buitrago et al., 2014), all training sessions were performed 60 min after ingestion of the pre-exercise supplement (30 g CHO + 15 g MCT). Warm-up sessions were also designed specifically to avoid fat oxidation and comprised 30 s bouts of intense exercise (at 80% of VO_2peak_ achieved in the incremental step test) before HIIT and sets of low repetitions at high speed (without weights) before strength training. With the same objective, we decided not to include a cool-down phase after HIIT sessions.

## Results

The effects of the training program are shown in Table 1. VO_2peak_ showed a high increase (90.2%), of which most (70.7%) occurred in the first 3 months. Also, peak values of HR and power output (W) increased 20.6 and 71.4%, respectively, in the first 3 months, without changes in the last 3 months.

**Table 1:** Ergospirometry data (peak values) during the laboratory assessments, before and after the combined (high-interval training HIIT + resistance training) exercise program.

Variables	Pretraining	Posttraining (3 months)	% Change (from pretraining to 3rd month of training)	Posttraining (6 months)	% Change (from pretraining to 6th month of training)
Test duration	13 min 45 s	23 min 40 s	+72.1	23 min 38 s	+71.9
VO_2peak_ (ml⋅kg^−1^⋅min^−1^)	11.6	19.9	+70.7	22.3	+90.2
VO_2peak_ (ml⋅min^−1^)	846	1441	+70.3	1610	+90.3
VCO_2_ (ml⋅min^−1^)	932	2,235	+139.3	1,714	+83.9
VE (ml⋅min^−1^)	27.3	73.5	+169.2	81	+196.7
VE/VO_2_	32	51	+59.4	50	+56.3
RER	1.10	1.55	+40.9	1.06	−3.6
HR (beats⋅min^−1^)	144	182	+26.4	180	+25
% HRmax	73	92	+26.0	91	+24.7
Power output (watts)	70	120^∗^	+71.4	120^∗^	+71.4

*^∗^Completed only 40 s of the last step. HR, heart rate; HR_max_, age-predicted maximum heart rate (220 minus age, in years); RER respiratory exchange ratio; VCO_2_ carbon dioxide production; VE, ventilation; VE/VO_2_ ventilatory equivalent for oxygen; VO_2peak_, peak oxygen uptake.*

**Table 2:** Aerobic and resistance training sessions averaged data during the combined training program.

	HIIT						Resistance training			
Month	HR (beats⋅min^−1^)	% of HR_max_	RPE	Pain (0 to 10 score)	Risk^∗^ (yes/no)	Swelling^∗^ (yes/no)	RPE_Legs_ (0 to 10 score)	RPE_Arms_ (0 to 10 score)	DOMS (0 to 10 score)	Swelling (yes/ no)
1	166 ± 13	85	2 ± 0	1 ± 1	No	No	–	–	–	–
2	154 ± 6	79	5 ± 1	0	No	No	2 ± 1	2 ± 1	2 ± 1	No
3	144 ± 12	73	4 ± 1	2 ± 0	No	No	1 ± 0	2 ± 0	2 ± 1	No
4	150 ± 8	77	3 ± 1	0	No	No	3 ± 0	4 ± 1	1 ± 1	No
5	138 ± 4	70	5 ± 0	0	No	No	5 ± 1	5 ± 1	3 ± 2	No
6	140 ± 4	70	5 ± 0	0	No	No	6 ± 0	5 ± 1	1 ± 1	No

*Data are presented as mean ± SD. DOMS, delayed onset muscle soreness (0–10 scale, one day after training sessions); HIIT, high-intensity interval training; HR, heart rate; HRmax, age-predicted maximum heart rate (220 minus age, in years); RPE, rating of perceived exertion (0–10 scale). ^∗^perception of swelling and of risk of actual muscle “crisis” (rhabdomyolysis) (yes/no) was reported 3 h after the end of training sessions.*

In HIIT training sessions, HR reached at the end of sets ranged between 70 to 85% of age-predicted maximum HR (220 minus age). Pain levels reported during the HIIT sessions were low and decreased during the months of training (Table 2). In addition, the patient did not perceive muscular swelling or risk of a crisis in the hours following each training session. The RPE in strength training increased during the training period, reaching the desired values in the 5th month. Delayed onset muscle swelling and soreness scores were low and none of the sessions caused her sense of muscular swelling (Table 2).

During the 6 months of supervised training the patient had no myalgia or contractures and did not report the presence of dark urine. In addition, and as opposed to the 2 years previous to the program (with one episode of rhabdomyolysis [CK = 24,860 U/L] requiring 3-day hospitalization in 2015 and two episodes in 2016, each requiring 4–day hospitalization [CK = 6,277 and 16,759, respectively]– Figure 1), blood parameters were in normal range during the training program, with CK values of 52, 55, and 169 U/L (months 0, 3, and 6, respectively), without any episode of rhabdomyolysis. The patient was not admitted to hospital during this period. The patient has remained active, performing similar but non-supervised training (including also pre-exercise MCT+CHO supplementation) for 1.5 years (to date). During this period, she has not experienced myalgia, contractures or rhabdomyolysis, and has not been admitted to hospital; she only visited the hospital once for a routine medical visit (in September 2018), showing a low CK value (=61 U/L). Similar to during the period of supervised training, she described less limitation in daily life activities and a higher quality of life.

## Discussion

The main finding of our study was that the combined (HIIT + strength training) exercise regimen with pre-exercise CHO + MCT supplementation yielded high benefits (i.e., ↑20.6% peak HR, ↑90.2% VO_2peak_, and ↑71.4% peak power output) for the patient. Our results also indicated that this regimen was safe, with no muscle pain, contractures, rhabdomyolysis (baseline CK < 200 U/L) or hospital admissions reported in the entire training period.

VO_2peak_ has been described as an indicator of cardiorespiratory health (Jones and Carter, 2000), morbidity (Levine, 2008) and mortality (Jones and Carter, 2000) in the general population. The improvement in VO_2peak_ can be attributed to multiple factors that increase aerobic fitness, including improvement of respiratory muscles, and both cardiovascular and peripheral factors (Vendrusculo et al., 2019). In this case, the improvement of VO_2peak_ could be explained by both peripheral (i.e., increased mitochondrial content of skeletal muscle and capillary density) and central cardiovascular adaptations (i.e., increased cardiac contractility, blood volume and cardiac output), widely described after HIIT (MacInnis and Gibala, 2017).

The training program and supplement were designed to prevent muscular discomfort during training, post-exercise muscle crises and increases in basal CK. Post-exercise rhabdomyolysis has been described in patients with VLCADD as a consequence of the impossibility in obtaining energy during exercise when it is performed close to FATMAX (Diekman et al., 2016). Although MCT supplementation can bypass the blockade in the oxidation of long chain triglycerides (LCT) and can provide a useful source of fatty acids for both the heart and the skeletal muscle in exercise (Behrend et al., 2012), it has been shown to have no benefits in patients with VLCADD when they exercise at FATMAX, when the energy demand of fats is very high (Ørngreen et al., 2007). However, the energy demand of LCT in the HIIT protocol used in our patient was much lower since skeletal muscle depends almost entirely on CHO as a source of fuel as exercise intensity increases (Van Loon et al., 2001), greatly reducing the energy demand of fats. Under these conditions, the MCT + CHO supplement seems to have been effective both during and after exercise. Indeed, CHO can be used as fuel during exercise (Behrend et al., 2012), increasing blood glucose levels and their use during and after exertion (Tsintzas et al., 2000). The sparing effects on the utilization of muscle glycogen stores, and the higher glycemia after exercise, seem to be responsible for the fact that the energy demand of fat oxidation during EPOC is moderate enough for MCT to be sufficient.

After the supervised training period, the patient maintained a similar training regimen until today, without experiencing muscular contractures or hospital admissions. The patient also reported improvement in her quality of life due to the relief from muscular discomfort. This could be due to the training adaptations, a better control of diet and a greater understanding of the disease. It is known that high pedaling cadences, high contraction speeds and low number of sets (such as those used in our HIIT sessions) produce muscle fiber type shifts characterized by an increase of type IIA fibers and a decrease of type I fibers (Wilson et al., 2012). Since type IIA fibers are more dependent on CHO metabolism and less on LCT metabolism (Egan and Zierath, 2013; Bourdeau Julien et al., 2018), a higher proportion of type IIA would allow an increase in the use of muscle glycogen and a decrease in the demand for LCT. This would reduce the limitations and the risk of muscle crises in the patient. Indeed, this metabolic adaptation (although without fiber type shift) has already been described in VLCAD^−/−^ mice as a strategy to compensate for VLCAD deficiency (Tucci et al., 2012). It is also known that strength training, similar to that used here, has proven to reduce rhabdomyolysis in other myopathies (Santalla et al., 2014; Pietrusz et al., 2018) as a consequence of an improvement in the resistance of muscle fibers to damage during physical activity. In addition, less muscle damage could allow for the maintenance of higher exercise intensities without experiencing symptoms (Pietrusz et al., 2018) and would imply a lower energy expenditure, from CHO and LCT, for the synthesis of muscle tissue during EPOC.

The patient followed a diet high in protein and low in fat during and subsequent to the intervention period. A diet regimen low in LCT is recommended for patients with VLCADD, but the necessary intake of essential fatty acids should be provided (Spiekerkoetter et al., 2009). Although this diet was prescribed after diagnosis (before the training period), in the 1st visit to our laboratory we explained to the patient the importance of diet and of avoiding fasting. She was also informed about the influence of exercise intensity and CHO availability on muscle fuel utilization during exercise and recovery. Relating to her own medical history, the patient was informed about how prolonged low intensity exercise performed during fasting caused rhabdomyolysis and hospital admissions. In the same way, the patient was instructed to carry out daily life activities at a higher speed and explosiveness, to avoid the energetic demand from oxidation of fatty acids (Egan and Zierath, 2013; Bourdeau Julien et al., 2018).

There are several limitations of this study that merit attention. As a single case report, the results must be interpreted with caution and cannot be generalized to other patients with VLCADD. In addition, gains in strength, quantitative changes (i.e., dual-energy X-ray absorptiometry assessments) and qualitative changes in muscle mass (i.e., muscle biopsy) were not quantified. We believe that the training program is the main reason that the patient stopped experiencing rhabdomyolysis and hospital admissions. However, we cannot establish the precise degree of responsibility, since the dietary habits were also changed and the way of carrying out activities of daily life was modified. Finally, we did not assess the patient’s quality of life or ability to cope with daily life activities, which should be done in future research.

In summary, the results of this study show that it is possible to carry out a combined (HIIT + strength training) exercise program in patients with VLCADD safely (without muscle contractures or rhabdomyolysis) and to achieve high values of VO_2peak_ and cycling power output. In our opinion, this must be supervised by a fitness specialist given the need to control the loads of HIIT, the strength training load and the monitoring of muscle perception during and after each training session.

## Ethics Statement

Ethics committee of Hospital 12 de Octubre, Madrid. Written informed consent was obtained.

## Author Contributions

AH-O: writing the work. JF-L: acquisition, analysis or interpretation of data (training). CP: medical care, testing and medical tracking of the patient, and also contributing to critical revision of the work. AL: contributing to the analysis and interpretation of the data and critical revision of the work. AS: conception and design the word, writing contribution and critical review of it.

## Conflict of Interest Statement

The authors declare that the research was conducted in the absence of any commercial or financial relationships that could be construed as a potential conflict of interest.
